# Evaluation of real-time fluorescence sensors and benchtop fluorescence for tracking and predicting sewage contamination in the Tijuana River Estuary at the US-Mexico border

**DOI:** 10.1016/j.scitotenv.2024.175137

**Published:** 2024-07-31

**Authors:** Natalie Mladenov, Trent Biggs, Keyshawn Ford, Stephany Garcia, Yongping Yuan, Alexandra Grant, Elise Piazza, Elisa Rivera, Federick Pinongcos, Scott P. Keely, Callie Summerlin, Jeffrey A. Crooks, Douglas Liden

**Affiliations:** a Department of Civil, Construction, and Environmental Engineering, San Diego State University, 5500 Campanile Drive, San Diego, CA 92182, United States of America; b Department of Geography, San Diego State University, 5500 Campanile Drive, San Diego, CA 92182, United States of America; c US Environmental Protection Agency, Office of Research and Development, 109 T. W. Alexander Dr., Research Triangle Park, NC 27711, United States of America; d US Environmental Protection Agency, Office of Research and Development, 26 W. Martin Luther King Dr., Cincinnati, OH 45268, United States of America; e Tijuana River National Estuarine Research Reserve, Imperial Beach, CA 91932, USA; f United States Environmental Protection Agency-Region 9 Water Division, San Diego, CA, United States of America

**Keywords:** Pollution, Wastewater, Tryptophan, Fluorescence, Coastal, Telemetry

## Abstract

Cross-border flow of untreated sewage from Mexico into the USA via the Tijuana River is public health issue with negative consequences for coastal communities. Here we evaluate the potential application of fluorescence-based, submersible tryptophan-like (TRP) and humic-like (CDOM) fluorescence sensors for real-time tracking of wastewater pollution in an estuarine environment. Sonde fluorescence measurements were compared with benchtop fluorescence, fecal indicator bacteria (FIB) concentrations, and real-time specific conductivity measurements in the Tijuana River Estuary during dry and wet weather conditions, and with and without cross-border flow. TRP and CDOM fluorescence concentrations were low during times without cross-border flow and two-three orders of magnitude higher during storm events and after cross-border sewage flow events. Major deterioration in water quality, including hypoxic conditions, was observed after consistent, long-term cross-border sewage flow. Real-time TRP and CDOM fluorescence concentrations had a significant linear relationship with fecal indicator bacteria (FIB) concentrations during dry weather periods with cross-border flow (*p* < 0.001) but were poorly correlated during stormflow and during less polluted periods with no cross-border flow. TRP and CDOM fluorescence acquired on discrete samples using a benchtop fluorometer correlated significantly (*p* < 0.001) with FIB concentrations under all cross-border flow conditions. Based on relationships between benchtop TRP fluorescence and percent wastewater, the greatest amount of untreated wastewater in the estuary’s surface layer during cross-border flow events was estimated at >80 % and occurred during neap tides, when concentrated, sewage-laden freshwater flowed over dense saline seawater due to stratification and lack of mixing in the estuary. These results are important because exposure to untreated sewage poses severe health risks for residents and visitors to adjacent coastal areas. While benchtop fluorescence was more effective for estimating the degree of wastewater pollution, submersible TRP and CDOM sensors provided a real-time alert of sewage contamination, which can be utilized in other sewage impacted estuarine environments.

## Graphical Abstract

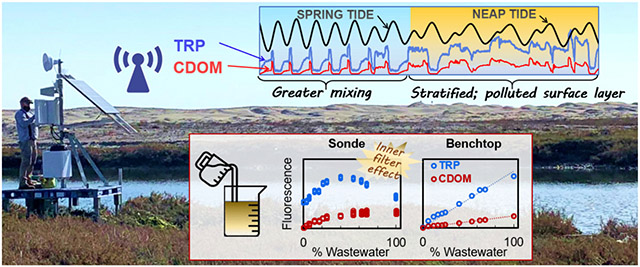

## 1. Introduction

Globally, >48 % of wastewater enters the environment untreated (Jones et al., 2021), making sewage contamination a genuine concern for coastal communities worldwide. Inadequate and failing sewer infrastructure combined with both wet weather and dry weather runoff has resulted in increased sewage contamination of the Tijuana River, a transboundary river that flows through Tijuana, Mexico, and northward into the USA where it discharges to the Pacific Ocean. The cross-border flow of sewage via the Tijuana River has been a litigious occurrence because it poses a public health risk for recreational beach visitors (Venn, 2021; Stigler Granados et al., 2024) and regularly reaches coastal areas near the outlet of the Tijuana River Estuary, resulting in extensive beach closures south of San Diego, CA, USA.

Sewage contains a range of fluorescent constituents, from microbial proteins and amino acids to fluorescent detergents and pharmaceuticals (Baker et al., 2015; Wasswa et al., 2019). Wasswa et al. (Wasswa et al., 2019) and Mendoza et al. (Mendoza et al., 2020) conducted organic chemical addition and wastewater addition experiments, respectively, in a controlled laboratory setting and showed that tryptophan-like (TRP) fluorescence and humic-like (CDOM) fluorescence sensors in portable, submersible fluorometers significantly correlated with pollutant concentrations. In natural riverine systems, fluorescence-based submersible sensors have been used for monitoring sewage and organic contaminants (Baker et al., 2015; Corsi et al., 2021); however, to our knowledge, the performance of fluorescence-based, in-situ sensors for tracking sewage contamination in estuarine conditions with a tidal influence has not yet been evaluated.

In recent decades, Tijuana River flows are often dominated by untreated sewage from the City of Tijuana, with treated wastewater effluent, groundwater, and stormwater runoff also contributing to the total flow (HDR, 2020). When flowrates in the Tijuana River exceed 1.0 m^3^/s, the volumes are too high to be routed to the International Water Treatment Plant (IWTP), which has a 1.0 m^3^/s diversion capacity (Arcadis U.S., Inc (Arcadis), 2019), leaving untreated sewage to flow through the Tijuana River channel uninterrupted and enter the Tijuana River National Estuarine Research Reserve (TRNERR) and pollute adjacent coastal communities.

To evaluate the ability of in-situ sensors to respond to sewage pollution in the TRNERR, we compared high-frequency (10-min), real-time fluorescence data from a submersible sonde equipped with TRP and CDOM fluorescence sensors to fecal indicator bacteria (FIB) concentrations in corresponding discrete samples collected during times with and without cross-border flow. In addition, fluorescence intensities acquired from a benchtop fluorometer capable of correcting for inner-filter effects were compared with sonde fluorescence results to evaluate the influence of light absorbing compounds on real-time sensor performance. We also evaluated water quality under spring and neap tide conditions and developed a simple model using fluorescence measurements to estimate the % sewage in the stratified water column.

## 2. Methods

### 2.1. Study site and sonde deployment

**Fig. 1.**
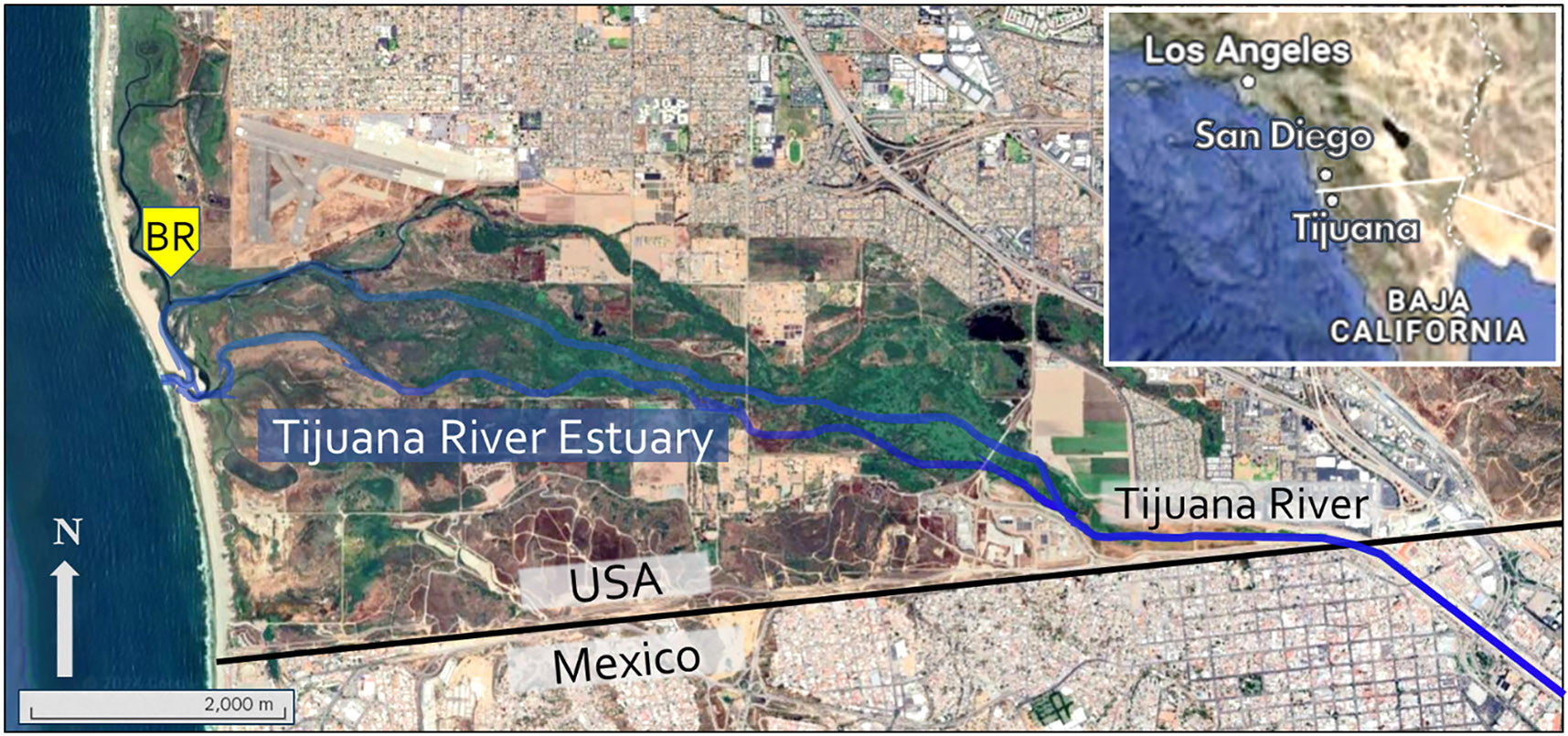
Map showing location of the Boca Rio sampling site and sensor platform (yellow “BR”) in the Tijuana River Estuary and the downstream reach of the Tijuana River.

The Tijuana River is a transboundary river that flows from Tijuana, Mexico, into the Tijuana River Estuary in the United States near the City of Imperial Beach and discharges into the Pacific Ocean. The watershed has a total area of 1750 mi^2^. A floating sensor platform was installed in the TRNERR at the Boca Rio sampling location (32.559597° N, 117.128765° W; Fig. 1), including a Manta multi-parameter sonde with fluorescence sensors for tryptophan (Turner Designs 2300–256, emits at 350/55 nm), CDOM (Turner Designs 2300–251, emits at 450 nm and chlorophyll *a* along with other sensors for turbidity (side-scatter (90^0^) Turner Designs SN 1901, ISO 7027 sensor type), pH, specific conductivity, temperature, dissolved oxygen (DO), and redox potential. The sonde had a brush that wipes all sensors before each measurement to prevent biofouling. The sonde was housed in a 4” diameter PVC tube, with copper mesh for additional biofouling protection. Data were logged and transmitted in real-time using the WQDataLive service.

Sensor maintenance and quality control of real-time, submersible fluorescence data is the focus of a companion study. In brief, the Manta sonde and sensor lenses were cleaned every ~2 weeks to reduce biofouling. Copper mesh housing was installed to reduce sensor maintenance visits from bi-weekly to monthly. Monthly maintenance also included checking that the housing was unobstructed by seaweed and floating litter, the central sensor wiper was functioning, and the buoy was properly positioned in the channel. Fluorescence sensors were calibrated periodically (1–3 month intervals). Some extended gaps in monitoring (sometimes weeks or months long) occurred when conditions were unsafe for sonde retrieval for calibration or maintenance. The data shown here were re-calibrated using tryptophan and CDOM standards. TRP standards (100 ppb) were prepared using dry tryptophan-L powder (CAS 73–22-3, Fisher Scientific bioreagents) and the TRP sensor was also checked against a solid standard. A quinine sulfate standard (300 ppb prepared in 0.1 N sulfuric acid) was used to calibrate the CDOM sensor, as per manufacturer recommendations, which was also checked against a solid standard. For TRP, the raw value (RV) was consistent over time, but sometimes departed significantly from the long-term mean, which could be due to error in standard preparation on that date. To account for such errors, we also calculated the RV for each TRP measurement and calculated a standardized TRP value using the long-term mean of the RV at 0 and 100 ppb TRP over the deployment period. AMCO polymer bead turbidity standards of both 100 and 1000 FNU (for ISO 7027 probes) were used to check and calibrate the turbidity sensor.

### 2.2. Sample collection

Stormwater samples were collected at the Boca Rio site during different hydrologic conditions (Table 1) at hourly time intervals over 24 h periods using a Teledyne ISCO 6700 autosampler. Six sampling events were conducted (Table 1) including: 1) dry weather conditions with no cross-border flow (referred to as “dry-no-flow”), 2) dry weather with cross-border flow (referred to as “dry-with-flow”), and 3) during and after a storm event with cross-border flows (referred to as “storm-flow”). The estuary receives both spring (semi-diurnal) and neap (mixed semi-diurnal) tides, and the tide types are also listed for each event in Table 1.

The ISCO intake tube was attached to the sonde housing with zip ties and retrieved samples from a depth to the sonde depth. Autosamplers were loaded with autoclaved (at 121 °C for 90 min) 1-L polypropylene (PP) bottles. Ice packs were placed in the interior chamber to chill autosampler bottles and minimize the potential decay of microorganisms and other constituents. As soon as samples could be safely retrieved, autosampler bottles were offloaded, transported to the lab, and immediately analyzed for fecal indicator bacteria (FIB) concentrations and basic water quality parameters (pH, specific conductivity, total suspended solids, and turbidity). For four of the six sampling campaigns, benchtop fluorescence spectra were acquired and dissolved organic carbon (DOC) and total dissolved nitrogen (TDN) concentrations were measured. Pre-event and post-event samples and field blanks (consisting of ultrapure water transferred to bottles in the field) were collected in autoclaved 1-L PP bottles as grab samples and immediately transported to the lab for analyses. Sample collection, preservation, storage conditions, and holding times are provided in Supplemental Table S2. Due to occasional dislodging of autosampler tubing, some autosampler bottles did not fill, resulting in times without sample collected. The number of processed samples for each site and storm event is listed in Table 1.

### 2.3. Wastewater addition experiments

Untreated wastewater was collected on several dates in 2022 and 2023 from an influent line at the International Water Treatment Plant (IWTP) in San Ysidro, CA, which receives wastewater primarily from the City of Tijuana. One untreated wastewater sample was collected from the San Elijo Water Reclamation Facility in San Diego County, USA. Additional water quality information for each wastewater sample is given in Supplementary Table S1. On the same dates, Pacific Ocean seawater was collected from the shoreline at Cardiff State Beach near Encinitas, CA (33.001509° N, 117.278600° W) in an area not impacted by Tijuana River pollution nor other nearby wastewater discharges, approximately 65 km north of the TRNERR. Wastewater addition experiments were performed in two ways, 1) by adding well-mixed wastewater incrementally to seawater in an opaque 120 L container containing portable, submersible Manta sondes with TRP and CDOM sensors for the real-time measurement or 2) by creating ~500 mL volumes at dilutions ranging from 0 % to 100 % wastewater and measuring the fluorescence of the solutions. The Manta sonde deployed at the Boca Rio site (#4105) was used in the lab for three wastewater addition experiments and other models were used in experiments when the #4105 sonde was in use at the sensor platform. In between each wastewater addition (for approach 1), the wastewater and seawater were hand-mixed for one minute. For approach 2, solutions were well mixed prior to analysis in the calibration cup. The sondes continuously logged fluorescence data and temperature for ~2 min at two-second increments. Samples were taken between each mixing step and filtered through glass fiber filters (0.7 μm) for fluorescence analysis (as described below) on the benchtop Horiba Aqualog fluorometer.

### 2.4. Chemical and optical spectroscopic analyses

In the lab, an Accumet AP85 Portable pH/Conductivity meter was used to measure pH, conductivity, and total dissolved solids (TDS) on unfiltered field samples. Field and experiment water samples were filtered (0.7 μm glass fiber filter, pre-combusted at 500 °C for 2 h) and analyzed for dissolved organic carbon (DOC; measured as non-purgeable organic carbon) and total dissolved nitrogen (TDN) using a high-temperature combustion method with a Shimadzu TOC-L Analyzer. Samples were either analyzed immediately after sample collection or acidified to between pH 2 and 3 with concentrated hydrochloric acid (37 %) for preservation until analysis. Triplicate measurements were performed for 10–15 % of the samples, and the standard deviations of replicates were within 10 % of the mean.

For filtered and unacidified field and experimental samples, three-dimensional excitation emission matrix spectra (EEMs) and UV–vis absorbance (used for inner filter correction) were acquired simultaneously using an Aqualog Fluorometer with a quartz cuvette (path length of 1 cm) and a 0.25 s integration time. Other instrument settings and corrections (blank subtraction, Raman normalization, and Rayleigh masking) are described in Mendoza et al. (Mendoza et al., 2020). The intensities of the tryptophan (TRP) and humic peaks (CDOM) that matched the peak wavelengths of the TRP and CDOM sensors, respectively, on the Manta sonde were recorded in Raman units (RU) and converted to ppb TRP (1 RU = 50.8 ppb TRP) and ppb CDOM (1 RU = 143.4 ppb CDOM) using calibration curves with tryptophan and quinine sulfate standards, respectively (Supplemental Fig. S8).

### 2.5. Microbiological analyses

Fecal indicator bacteria (total coliforms, *E. coli*, and enterococci) were analyzed in unfiltered water samples within 6 h of offloading autosamplers and within 24 h of collection for most events. Concentrations of FIB in a 100 mL volume were quantified using the IDEXX Colilert-18 and Enterolert methods, following the manufacturer’s recommended protocols. Samples underwent serial dilution and Quantitrays containing sample and reagent were sealed and immediately placed in an incubator at 35 ± 0.5 °C for 18 h (for Colilert-18) and at 41 ± 0.5 °C for 24 h (for Enterolert). Counts of positive wells were converted to final bacteria concentrations (MPN/100 mL).

### 2.6. Estimation of % wastewater in the Tijuana River Estuary

Due to the greater sensitivity of the benchtop fluorometer, fluorescence of discrete samples was utilized to predict % untreated wastewater in a stratified surface layer of the Tijuana Estuary. Relationships between % wastewater in our wastewater addition experiments and TRP-like fluorescence acquired with the benchtop fluorometer gave an estimate of % wastewater in the stratified surface layer for each snapshot sampling campaign as follows:

(1) $$\%\text{wastewater}=F_{\text{TRP}}∕k_{\text{avg}}$$

where $F_{\text{TRP}}$ is the TRP-like fluorescence intensity measured using the benchtop fluorometer (in RU) and $k_{\text{avg}}$ is the average slope of the linear relationship between % wastewater and TRP fluorescence for five different experiments of wastewater addition to seawater. It is important to note that this estimation is based on experimental results using untreated wastewater and that treated effluent would have different TRP and CDOM fluorescence and different $k_{\text{avg}}$ values. In addition, untreated wastewater used in the experiments differed in strength and quality from day to day; wastewater quality changes both diurnally (e.g., higher strength “brown wave” after morning water usage) and seasonally (e.g., Almedeij and Aljarallah, 2011; Abou-Elela et al., 2016; Mladenov et al., 2018). Therefore, the slopes of the linear regression lines between TRP fluorescence and % wastewater for 5 different wastewater addition experiments ranged from 0.07 to 0.19, with 0.16 as the average. We also evaluated whether correlations between benchtop fluorescence and DOC concentrations or chemical oxygen demand (COD) concentrations of wastewater (instead of % wastewater) for the 5 experiments would result in more reproducible linear regression slopes, but the slopes of those relationships varied even more widely than the % wastewater relationships.

### 2.7. Statistical analyses

IBM SPSS Statistics ver 29.0.1.0 was employed to evaluate the significance (95 % confidence intervals) of two-tailed Pearson bivariate correlations between variables.

## 3. Results and discussion

### 3.1. Long-term monitoring of sewage signal in the estuary

**Fig. 2.**
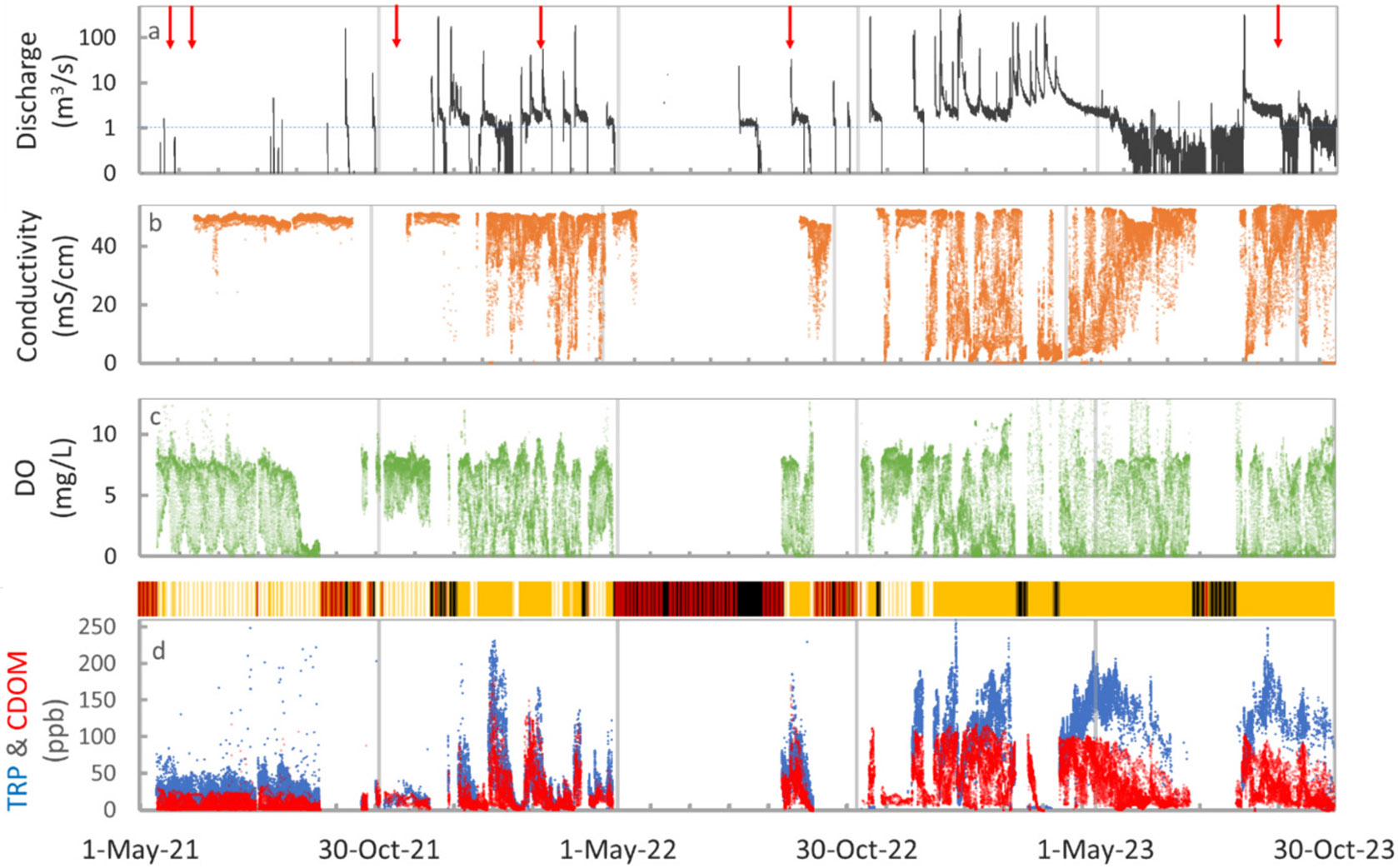
Time series of (a) discharge in the Tijuana River at the US-Mexico border (on log scale), (b) specific conductivity, (c) dissolved oxygen (DO) concentrations, and (d) TRP (blue) and CDOM (red) fluorescence concentrations available from the multiparameter sonde at the Boca Rio site. Red arrows in (a) show the dates of field ISCO sampling between May 2021 and October 2023. Gold bars in (d) show periods with cross-border flow; black bars and dark red bars show maintenance dates (with and without cross-border flow, respectively) when fluorescence sensor data were not recorded; white bars show periods without cross-border flow. Dashed line in (a) denotes the 1 m^3^/s discharge, above which river flows are not intercepted by the IWTP. DO, TRP, and CDOM concentrations corresponding to high tide conditions (with conductivity >50 mS/cm) are excluded for clarity.

Over the study period (May 2021 – October 2023), the sensor platform at the Boca Rio site in the Tijuana River Estuary provided reliable 10-min real-time water quality data transferred via telemetry. Frequent maintenance was required to calibrate and clean the sensors to reduce biofouling, and the maintenance periods, when no data were available, are shown in black and red bars in Fig. 2. A large gap in the data series occurred from May to Sep 2022, when the sensor was removed for repair and recalibration, but ~12 months of near-continuous data prior to the repair period and ~ 12 months after the repair period were still collected. A real-time camera co-located at the site was advantageous for detecting issues with the platform.

During the first two years of the study, there were extensive periods without cross-border flow (Fig. 2). Normally, river flows <1 m^3^/s are pumped to the IWTP for wastewater treatment, but frequent pump breakdown and other infrastructure failures result in sewage flowing across the border uninterrupted. In addition, due to the limited capacity of the IWTP, when discharge exceeds the 1 m^3^/s value, water is not pumped to the IWTP and all river flow continues across the border. From Dec 2021 to May 2022 and from Jan 2023 to Oct 2023, discharge frequently exceeded the 1 m^3^/s value, and cross-border flows were a regular occurrence. Notable differences between periods of infrequent cross-border flow (e.g., May 2021 to Dec 2021) and persistent cross-border flow were reflected in the real-time sensor data. Persistent cross-border flow resulted in longer durations with low DO concentrations and high TRP and CDOM fluorescence, characteristic of sewage contamination. Peak TRP and CDOM fluorescence concentrations during the persistent cross-border flow periods increased by ~80 ppb and ~50 ppb, respectively, compared to periods with infrequent cross-border flow and were sustained for longer durations (Fig. 2). In general, TRP concentrations were higher than CDOM concentrations, reflecting the greater TRP fluorescence associated with sewage (Baker et al., 2015).

### 3.2. Tracking of microbial pollution under different hydrologic conditions

**Fig. 3.**
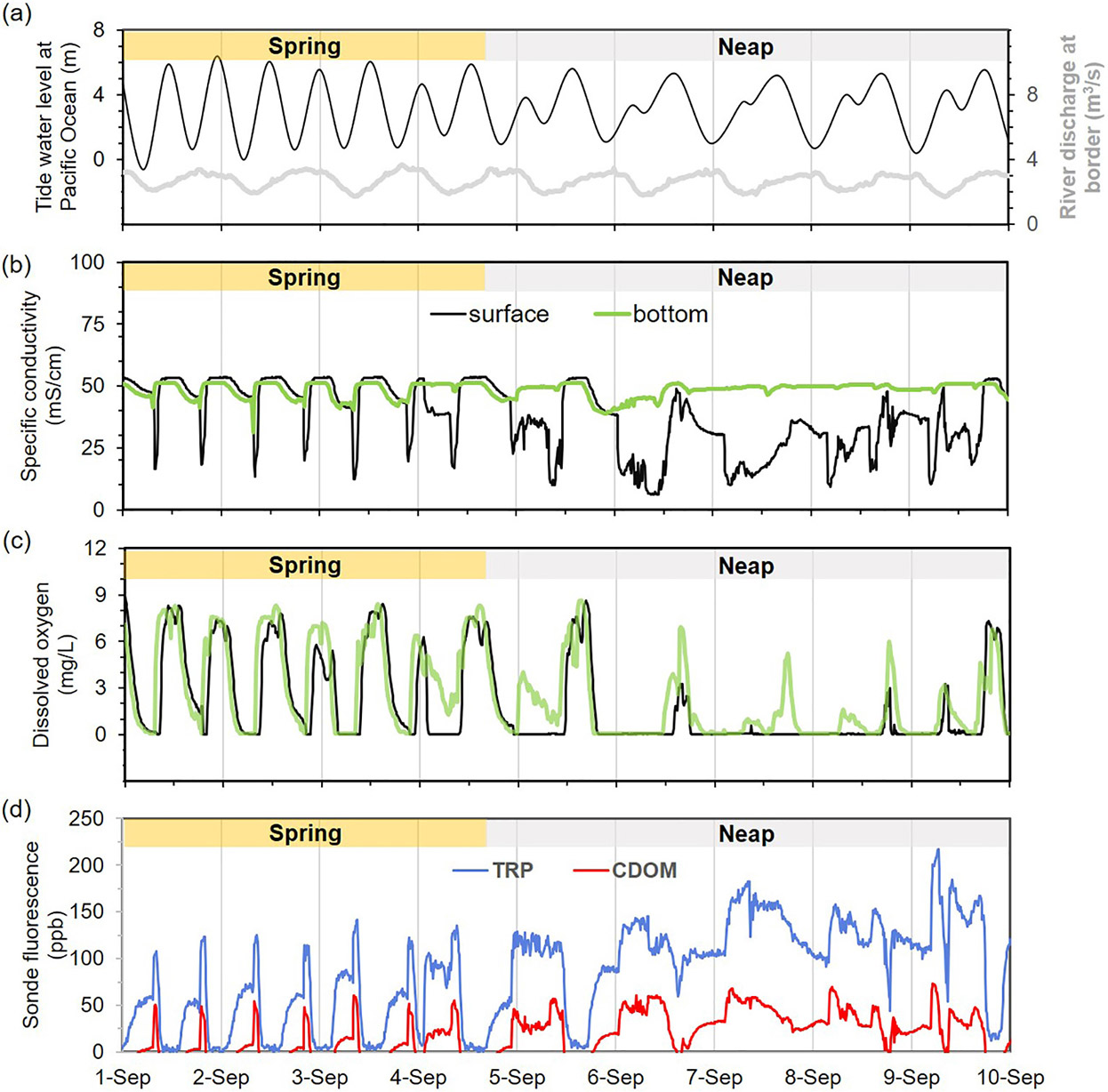
a) Tide water level in the Tijuana River Estuary (black) during spring and neap tides at San Diego, California from a representative rain-free period from 1 to 10 September 2023 and Tijuana River discharge (gray) at the border for the same period. Response of water quality parameters b) specific conductivity and c) dissolved oxygen measured using the Manta sonde (at 20 cm below water surface) and TRNERR sonde (fixed to estuary bottom), and d) TRP (blue) and CDOM (red) fluorescence measured using the Manta sonde. Approximate spring tide and neap tide periods are labeled. Discharge in the Tijuana River at the US-Mexico border fluctuated daily, but remained between 2 and 4 m^3^/s for the period shown.

We found that spring tides (high tidal amplitude) and neap tides (low amplitude) characteristic of the US West Coast (Nidzieko, 2010) also had an important influence on pollution levels in the estuary. Examples of spring tide and neap tide conditions are shown in Fig. 3 for a representative rain-free period during 1–10 September 2023 (Fig. 2). Overall, high tide coincided with the highest conductivity (reaching >53 mS/cm for Pacific Ocean water), highest dissolved oxygen concentrations, and lowest fluorescence values (Fig. 2), indicating river wastewater dilution by incoming seawater. Meanwhile, the lowest water levels (low tide) had the lowest conductivity, lowest DO, and highest fluorescence values, reflecting the contributions from the sewage-contaminated Tijuana River as it enters the Tijuana Estuary.

Spring tides during 1–4 September 2023 resulted in greater estuary mixing (Vörösmarty and Loder, 1994) and wide swings in specific conductivity and dissolved oxygen. Similar specific conductivity and DO concentration were measured with both the Manta sonde, which is deployed from a buoy at ~20 cm just below the water surface, and the deeper TRNERR sonde (YSI EXO2), which is fixed 0.5 m off the estuary bottom. These similarities reflect a more well mixed estuary water column during spring tide (Vörösmarty and Loder, 1994). By contrast, specific conductivity measurements diverged for the two sondes during neap tide conditions (5–10 September 2023 in Fig. 3b), indicating a stratified water column, with lower salinity river water poised above denser saline ocean water. Near the surface, conductivity never reached >50 mS/cm during neap tides, meaning that inputs of seawater into the estuary were suppressed and that Tijuana River water dominated the estuary. Dissolved oxygen reached its lowest values for both sondes during neap tides, and completely anoxic conditions were present during most of the neap tidal cycle (Fig. 3c).

During spring tide conditions, TRP fluorescence (measured only near the water surface) exceeded 100 ppb during the short transition from low tide to high tide and remained low during high tides (Fig. 3d). Neap tide conditions produced a longer sewage-derived TRP signal with minimal dilution by seawater. TRP fluorescence exceeded 100 ppb during most of the neap tide cycle and even doubled (reaching >200 ppb) on 9 September 2023. The temporal variability in CDOM was similar to that of TRP. CDOM peaked at values between 50 and 75 ppb and remained elevated for longer durations during neap tides than during spring tides.

**Fig. 4.**
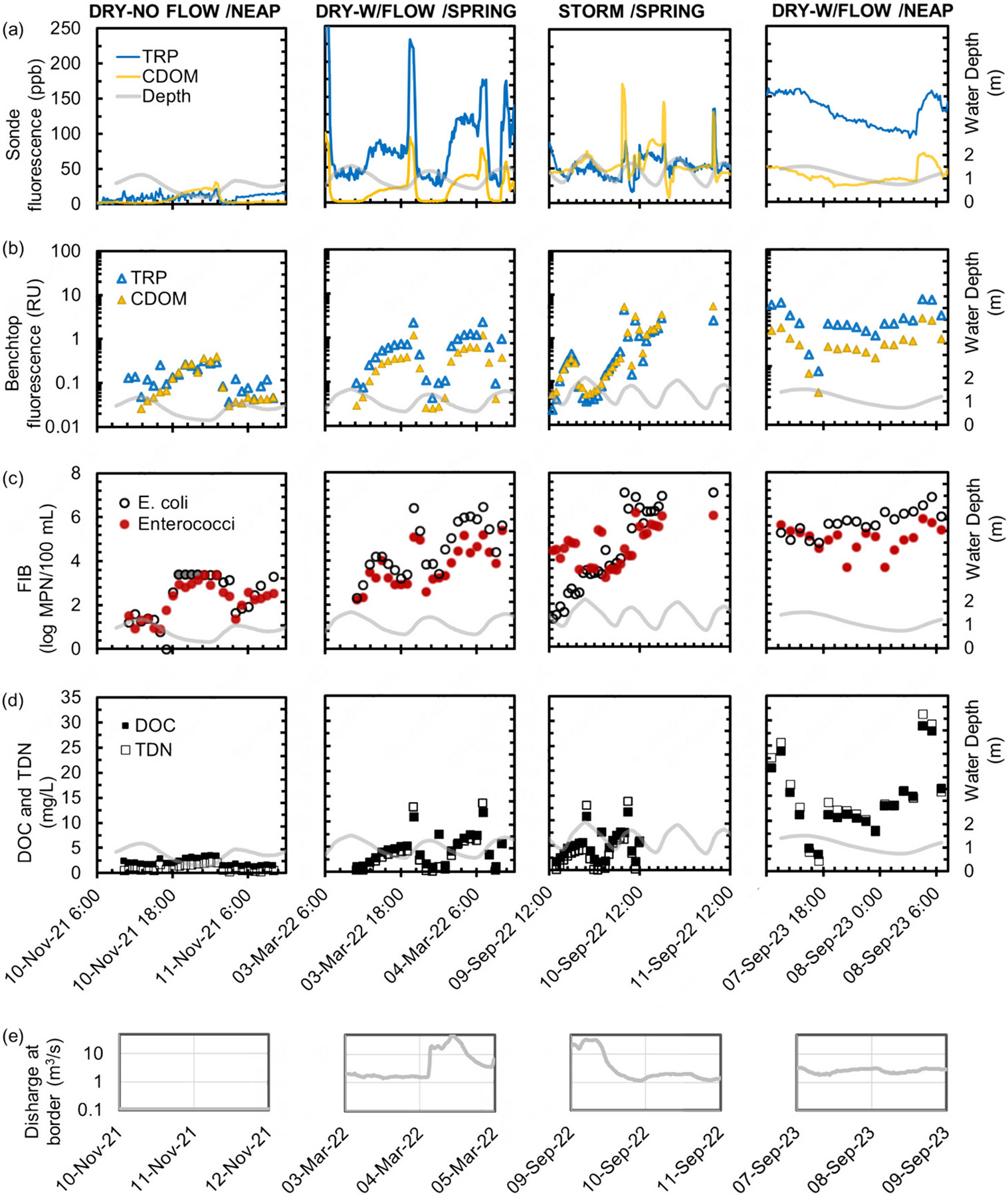
Representative time series showing changes in water level (right y-axis) and a) TRP and CDOM fluorescence obtained from the submersible sonde, b) TRP and CDOM benchtop fluorescence, c) *E. coli* and enterococci concentrations, and d) DOC and TDN concentrations at the Boca Rio site under spring tide and neap tide conditions, with and without cross-border flow. Panel (e) shows discharge on a log scale at the US-Mexico border during the sampling events. Gray shaded circles in the 10–11 November 2021 event represent values that exceeded the maximum concentrations of the dilution used in the IDEXX Colilert test, respectively.

Discrete samples were also collected by autosampler with tubing located at a similar depth to the Manta sonde, representing water of the near-surface zone. Data from four of the six campaigns are shown in Fig. 4; benchtop fluorescence and DOC and TDN measurements were not available for the first two campaigns. For all sampling campaigns, the lowest FIB concentrations occurred during high tides, whereas low tides had elevated FIB concentrations. The tidal influence was dampened or not as evident for samples collected during the storm event (September 2022), which may be due to runoff-driven Tijuana River discharge having an overriding effect on the incoming tide and other source areas (e.g., cross-border runoff flowing through canyon collectors and into the estuary).

During the neap tide and storm events, FIB concentrations (with total coliforms reaching >10^7^ MPN/100 mL and *E. coli* and enterococci both reaching >10^6^ MPN/100 mL; Fig. 4 and Supplemental Figs. S1 - S3) were orders of magnitude higher than during dry-no-flow conditions and similar to those of untreated wastewater from the City of Tijuana at the IWTP, which were on the order of 10^7^ MPN/100 mL for *E. coli* and 10^6^ MPN/100 mL for enterococci during our wastewater addition experiments. The highest DOC and TDN concentrations (both exceeding 25 mg/L) were also measured during the September 2023 neap tide event, and are evidence of the tremendous efflux of organic matter from the Tijuana River into the estuary. Bacterial degradation of organic matter and nitrification of ammonium both consume oxygen and would be expected to contribute to hypoxia, which is reflected in the low DO concentrations already observed in the estuary, especially during neap tides (Fig. 3c). Residents of the City of Imperial Beach report long-term foul odors every few weeks (*E. Luna*, 2024, personal communication), which may be a consequence of low DO and particularly poor water quality during neap tide events.

The extremely elevated FIB concentrations during the dry-with flow/neap tide event (right panel of Fig. 4c) further demonstrate the intense sewage pollution during weak estuarine mixing by neap tide conditions. Observations and models from other estuarine studies also show that neap tide conditions result in less mixing, greater stratification, and higher organic matter and nutrient concentrations compared to spring tide conditions (Vörösmarty and Loder, 1994; Nascimento et al., 2021). Elevated pollutant concentrations exiting the estuary during neap tides may pose a further risk when the coastal area experiences alongshore currents, characterized by high-concentration plumes close to the shoreline (Rippy, 2012).

During the dry-no-flow sampling event (November 2021; left panel of Fig. 4c), maximum enterococci concentrations reached ~10^3^ MPN/100 mL and *E. coli* concentrations exceeded 10^3.4^ MPN/100 mL. There is currently no water quality objective (WQO) for the Tijuana River, but these values were well above the dry weather WQO for other waterways in San Diego County. For example, the 30-day geometric mean WQO is 200 MPN/100 mL for fecal coliform (of which *E. coli* is a member) and 35 MPN/100 mL for enterococci for the Upper San Diego River during dry weather (California Regional Water Quality Control Board San Diego Region (San Diego Water Board), 2019). Even without cross-border flow, FIB concentrations of water exiting the Tijuana River Estuary far exceeded typical regulatory thresholds for other rivers and highlight the extensive and persistent fecal contamination of the estuary.

### 3.3. Performance of fluorescence-based sensors

**Fig. 5.**
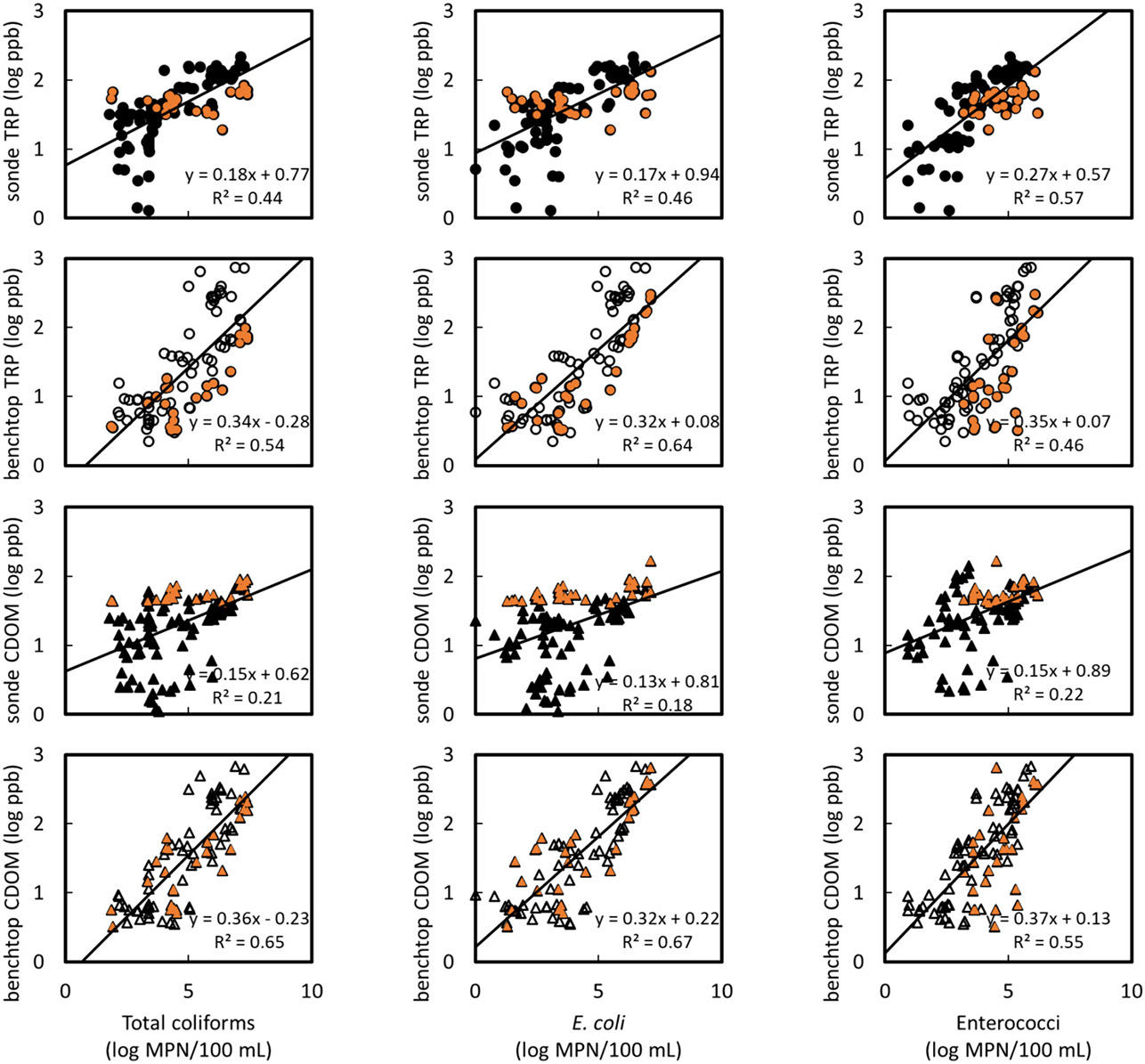
Relationships between fecal indicator bacteria (x-axis) and TRP-like (circles, top 2 rows) and humic CDOM-like (triangles, bottom 2 rows) fluorescence for all sampling events combined. Sonde (in-situ) fluorescence and benchtop fluorescence are shown with solid and unfilled black symbols, respectively. Orange colored symbols show data from only the stormflow sampling event. Relationships between FIB and both benchtop and sonde data were significant when all data are combined (*p* < 0.001).

In the current study, fluorescence sensor intensities are compared with FIB concentrations because FIB have long been used as surrogates for detecting human fecal pollution (Boehm et al., 2003) and indicate the presence of pathogenic microorganisms, which are of particular concern for coastal communities. For all events with cross-border flow, both CDOM or TRP fluorescence from the benchtop instrument (Fig. 4b) captured major increases or decreases in the FIB concentrations, demonstrating synchrony between benchtop fluorescence and *E. coli* and enterococci concentrations (Fig. 4). However, sonde fluorescence (Fig. 4a) was really only synchronous with FIB concentrations during the dry-with-flow event of March 2022. When fluorescence intensities from the six sampling events were combined and plotted against FIB concentrations, relationships between FIB and fluorescence concentrations were highly significant for TRP and CDOM from both the sonde and benchtop instruments *(p* < 0.001; Fig. 5). However, for individual events (Supplemental Figs. S1 - S6), correlations between FIB concentrations, sonde fluorescence, and benchtop fluorescence were less significant or, in some cases, non-existent. For example, samples collected during wet weather (stormflow event of September 2022), were poorly correlated to FIB concentrations (Fig. 5). Results from benchtop fluorescence, which is inner filter effect-corrected, indicate a greater % wastewater during this storm event, and suggest that the poor relationships between sonde fluorescence and FIB concentrations may be due to fluorescence quenching by the inner filter effect. The most significant relationships with both *E. coli* and enterococci occurred during the March 2022 dry- with flow event (Fig. S4), and these conditions may therefore be the most reliable for using the in-situ sonde for tracking sewage and microbial pollution in the estuary.

For most sampling events, FIB concentrations correlated more closely with TRP and CDOM intensities from the benchtop fluorometer than with TRP and CDOM from the in-situ sensor. The superior performance of the benchtop fluorometer compared to portable sensors is not unexpected, due to removal of interferences of turbidity by filtration and of instrument corrections, and has been reported in other studies (e.g., Mendoza et al., 2020). Nevertheless, both the in-situ TRP and CDOM sensor fluorescence had more significant correlations with FIB concentrations during more polluted conditions (Supplemental Figs. S4 - S6) and insignificant correlations during less polluted conditions, when FIB concentrations and fluorescence intensities are low (Figs. S1 and S2). In the latter case, background fluorescence from other DOM sources in the estuary may be present and impact these relationships. Recent laboratory experiments by our team (unpublished) also indicate that exposure to sunlight (photodegradation) and microbial decomposition in the dark (biodegradation) have diverging effects on FIB concentrations and fluorescence intensities when waters are dilute (20 % wastewater) than when they are higher strength and concentrated in colored organic matter (100 % wastewater).

### 3.4. Submersible and benchtop fluorescence sensor response to wastewater addition

**Fig. 6.**
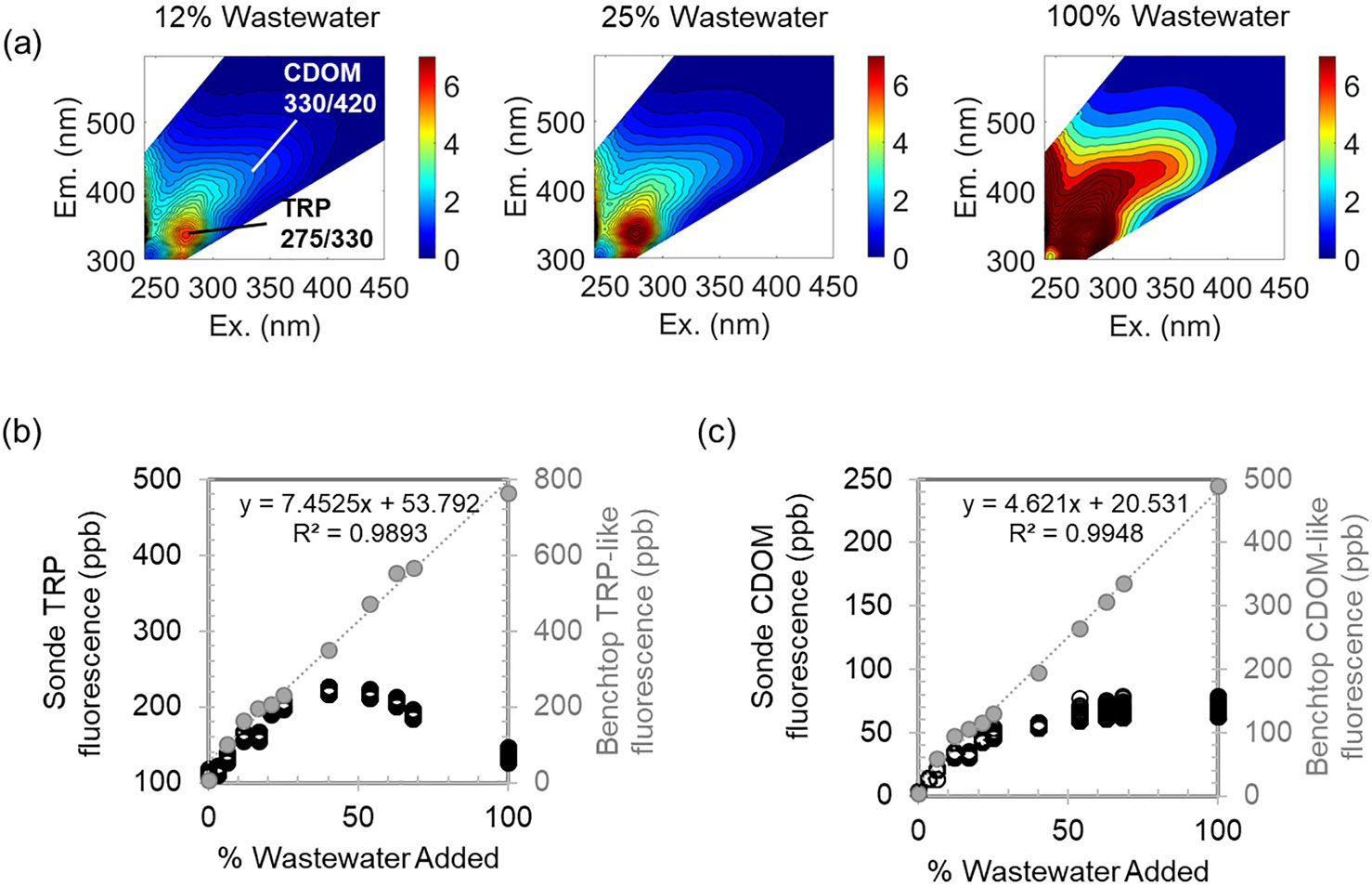
a) Representative EEM spectra from the benchtop fluorometer of untreated IWTP wastewater added to seawater at different increments, with TRP-like fluorescence (peak at ex/em of ~275 nm/330 nm) and CDOM fluorescence (peak at ex/em of ~330/420 nm) both increasing with increasing % wastewater added. Response of b) TRP and c) CDOM fluorescence to wastewater addition, measured using the submersible sonde (black circles) and the benchtop fluorometer (gray dots) for the 17 Nov 2022 wastewater addition experiment.

In addition to degradation effects on microorganisms and fluorescence properties of DOM described above, the presence of light-absorbing compounds inhibits fluorescence, particularly for portable, submersible sensors. Wastewater addition experiments were performed to test the capabilities and limitations of the portable, submersible sonde and the benchtop fluorometer to wastewater addition. Although wastewater contains humic DOM as well as proteinaceous DOM, the most prominent fluorescence in the Peak T and B regions (Coble, 1996) of excitation emission matrix (EEM) spectra derive from proteins and amino acids in wastewater (Baker et al., 2015). In our wastewater addition experiments, the evolution of the TRP-like fluorescence peak in the benchtop fluorometer’s 3D EEM spectra is observed as wastewater is added to seawater (Fig. 6a). The more pronounced and higher intensity TRP than CDOM fluorescence is known and expected for untreated wastewater, whereas after biological wastewater treatment the intensities of both the TRP and CDOM peaks are greatly reduced and the CDOM peak can be of similar magnitude as the TRP peak (Mladenov et al., 2024). Therefore, TRP fluorescence is appropriate for sensor-based tracking of untreated wastewater in natural waters.

As higher proportions of untreated wastewater are added, the solution transmissivity decreases and light-absorbing compounds prevent light emission from reaching the detector, resulting in first and second-order inner-filter effects, which can preferentially reduce fluorescence in the tryptophan region (Sgroi et al., 2020; Goffin et al., 2020). Previous experimental studies showing significant correlations between both amino acid-like (TRP-like) fluorescence and % untreated wastewater (Wasswa et al., 2019; Mendoza et al., 2020) only analyzed solutions containing <20 % wastewater. For wastewater addition up to ~25 % wastewater, we also observed a linear response from both of the TRP and CDOM measurement methods (benchtop fluorometer, for which samples were collected and filtered prior to analysis, and portable sonde, which was submerged in unfiltered solution); however, greater ratios of wastewater:seawater resulted in a decrease in portable TRP sensor intensities (Fig. 6b) and a plateau in CDOM sensor intensities (Fig. 6c), due to both inner-filter and turbidity effects. Upstream of the estuary at the international border, Tijuana River water has much higher turbidity, and in-situ TRP and CDOM fluorescence both have been observed to rarely exceed 100 ppb at the border due to turbidity and inner filter effects.

Post-processing of benchtop fluorometer EEMs with inner-filter effect corrections (Lakowicz, 1999; McKnight et al., 2001) and dilution reduce or eliminate the inner filter effect, resulting in a linear increase in fluorescence intensity with increasing % wastewater. Indeed, the intensities of both TRP and CDOM peaks in EEMs of filtered wastewater, acquired using the benchtop fluorometer were significantly correlated with percent wastewater (both with $R^{2}>0.99$; *p* < 0.001) for all five wastewater addition experiments (Fig. 6b and c and Supplemental Fig. S7), and did not plateau or decrease as wastewater content increased. Filtration of samples prior to analysis with the benchtop fluorometer also eliminates turbidity interferences, which act to expand the Rayleigh scatter bands and quench fluorescence (Khamis et al., 2015).

Inner-filter effect corrections are not yet possible for real-time, in-situ sensors, and this may be a drawback of fluorescence-based real-time monitoring of water bodies with very high wastewater pollution. Turbidity corrections are being developed for the Tijuana River in a companion study [Biggs et al., in prep] and already exist for other turbid environments (Goffin et al., 2020; Khamis et al., 2015). However, by the time water reaches the outlet of the Tijuana River Estuary, turbidity is low (< 150 FNU during all six sampling campaigns) and water parcels will have undergone both dilution with seawater and degradation during transport, which further reduce the amount of light-absorbing DOM and enable more accurate estimation of % wastewater.

### 3.5. Predictive capability of fluorescence-based sensors

**Fig. 7.**
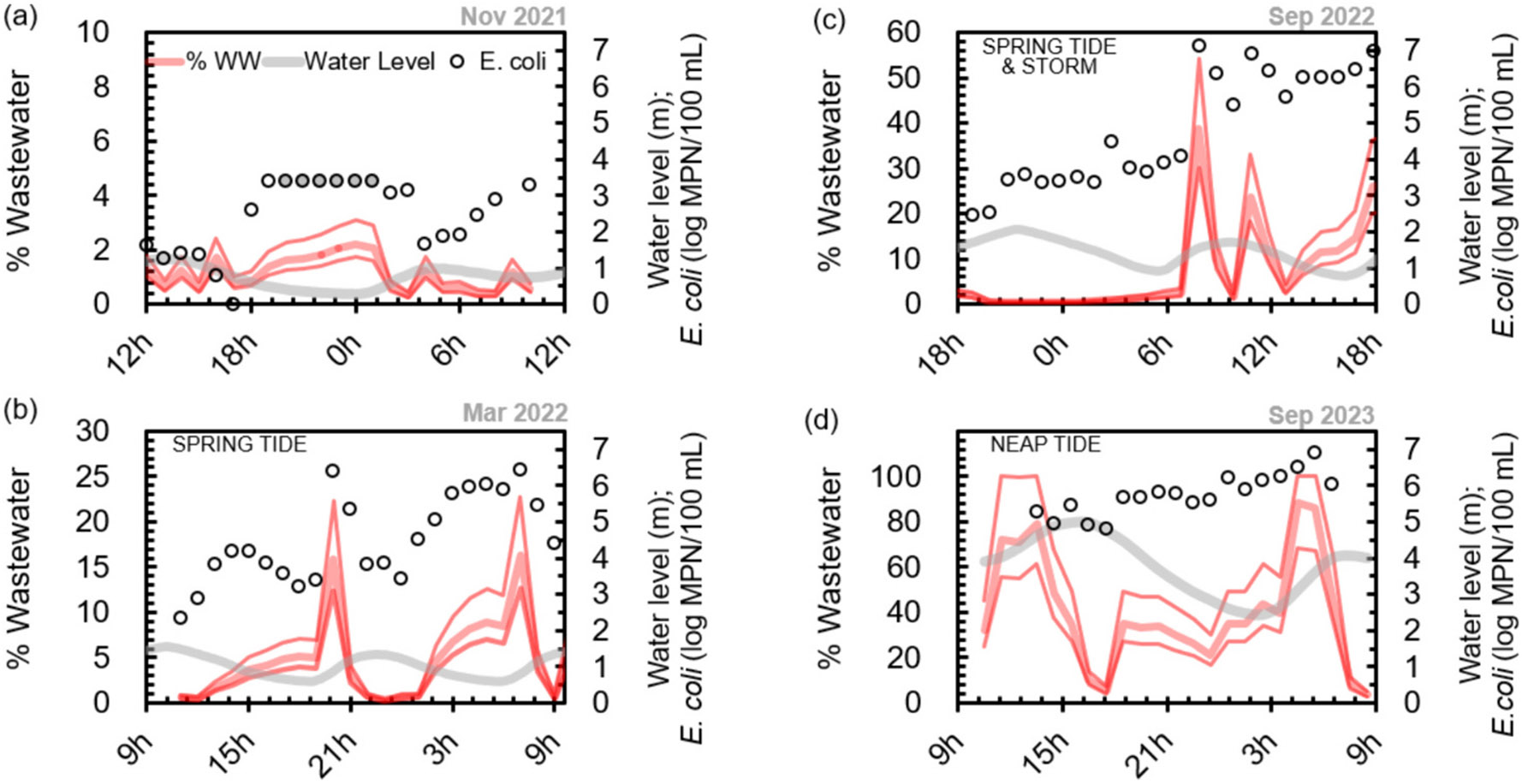
Estimated % wastewater (thick red line) and range (thin red lines) in the stratified surface layer of the Tijuana River Estuary calculated for: a) dry-no-flow (Nov 2021), b) dry-with-flow during spring tide (Mar 2022), c) stormflow (Sep 2022), and d) dry-with-flow during neap tide (Sep 2023) events. Corresponding water levels in the estuary and measured *E. coli* concentrations included. Shaded circle markers in (a) indicate *E. coli* concentrations exceeding 2420 MPN/100 mL, the maximum of the IDEXX Quantitray.

The average % untreated wastewater (and range) estimated for the estuary’s surface layer is plotted for four of the snapshot events (Fig. 7) with panels (a) no-flow, (b) with-flow-spring tide, and (d) with-flow-neap tide representing dry weather conditions and panel (c) representing stormflow conditions. The estimation was performed using only TRP fluorescence data acquired with the benchtop fluorometer. During the dry-no-flow event of November 2021, which occurred ~18 days after a storm event (Table 1), the percent wastewater was much lower (~ 2 %) than during storms or dry-with-flow events (Fig. 7). However, FIB concentrations still exceeded benchmarks (Fig. 4c), reflecting the persistence of fecal contamination in the estuary and its potential to contaminate the coastal zone. The lower TRP fluorescence and % wastewater during the peak of the high tide reflects dilution and improved water quality.

Higher % wastewater with longer duration occurs during storm events (e.g., September 2022) and neap tide events (e.g., September 2023). During the frequent dry-with-flow conditions that have been observed in the Tijuana River in recent years, there is a large range in the % wastewater of the stratified layer, which may be as low as 0 % just after a spring high tide (March 2022 snapshot; Fig. 7b) or as high as >80 % just after a neap low tide (September 2023 snapshot; Fig. 7d). *E. coli* concentrations, which exceeded 10^6^ MPN/100 mL during the neap tide event (Figs. 3d and 6d) further support these high estimates of wastewater in the Tijuana River Estuary.

The potentially lower % wastewater in the estuary’s surface layers during high spring tides as well as the poor water quality during neap tides are important for public and ecosystem health, though the connections between water quality in the estuary and the coast are not well documented. Recent modeling of coastal fecal pollution sources at the US-Mexico border, the Tijuana River outflow, and San Antonio de los Buenos outfall at Pt. Bandera, show that both sources impact swimmer illness risk (Feddersen et al., 2021). Pendergraft et al. (Pendergraft et al., 2023) further demonstrated that sewage-derived pollutants transfer in sea spray aerosol and may expose many people along the coast. Consideration of the greater pollution under neap tide conditions is important for understanding when increased bacteria may be present.

## 4. Conclusions

The response of TRP and CDOM fluorescence sensors to cross-border sewage flows was evaluated from May 2021 to October 2023 using a submersible, in-situ sonde deployed in the tidally-influenced Tijuana River Estuary. Overall, we found that, despite inner filter effects at high wastewater concentrations, submersible sensors of tryptophan-like and humic-like fluorescence were effective for distinguishing times with and without cross-border sewage flows and can provide critical warning of sewage pollution in an estuarine environment. However, there were limitations to the use of in-situ fluorescence-based sensors for estimating FIB concentrations, especially during periods without cross-border sewage flow, when FIB concentrations were low and variable. During periods when light-absorbing compounds are present at very high concentrations, such as during storm events or neap tide events, sensor data were subject to inner filter effects, which we confirmed with laboratory wastewater addition experiments. Frequent biofouling, maintenance and monitoring of sensor performance in calibration solutions were also issues for use of real-time water quality monitoring sensors. Due to the benchtop fluorometer’s more sensitive optics, inner filter effect corrections, and sample filtration prior to spectral acquisition, fluorescence peak intensities acquired using the benchtop fluorometer were more reliable surrogates of sewage pollution than those from in-situ sensors.

This study revealed a major deterioration in water quality after persistent, long-term cross-border sewage flow. In addition, neap tides were found to exacerbate wastewater pollution in the estuary, with stratification during this time resulting in the highest FIB concentrations, highest TRP and CDOM intensities, and lowest dissolved oxygen for the longest duration. The continuous cross-border sewage flow also delivered organic matter and nitrogen to the estuary, which appears to have prompted hypoxia, especially when tidal mixing was stunted under neap tide conditions. Cross-border sewage pollution is often a contested issue for transboundary communities worldwide; therefore, these findings provide important information about the extent of contamination in the Tijuana River Estuary and the utility of fluorescence-based sensors for other sewage-impacted estuary and brackish water environments.

## Supplementary Material

Supplement1

## Figures and Tables

**Table 1:** Dates of sampling events, antecedent dry days, rainfall characterization, and sampling information.

Event	Type	Date	Tide type	Antecedent dry days^a^	Rainfall during antecedent storm (mm)	Max discharge in Tijuana River during sampling (m^3^/s)	Turbidity (FNU)	Number of samples
1	Dry-no-flow	24–25 May 2021	spring	58	3.56^b^	0.0^c^	2.28	11
2	Dry-no-flow	6–7 Jun 2021	spring	71		0.0^c^	2.70	12
3	Dry-no-flow	11–12 Nov 2021	neap	16	6.86^b^	0.0^c^	2.72	24
4	Dry-with-flow	3–4 Mar 2022	spring	7	8.38^b^	55.2^c^	5.14	24
5	Stormflow	9–10 Sep 2022	spring	0	6.86^b^	33.1^c^	59.2	31
6	Dry-with-flow	7–8 Sep 2023	neap	17	35.81^b^	3.3^c^	14.9	18

a Number of dry days preceding the event with precipitation <2.5 mm.

b Recorded at Brownfield Municipal Airport National Oceanic and Atmospheric Association Weather Station in San Diego.

c Recorded by International Boundary and Water Commission at Tijuana River at International Boundary.

## Data Availability

Data will be made available on request.

## HIGHLIGHTS

- Real-time fluorescence sensor tracking sewage in an estuary was investigated.
- Sensor fluorescence values were elevated during cross-border sewage flow events.
- Benchtop tryptophan fluorescence with corrections had superior performance.
- Neap tide exacerbated poor water quality in the Tijuana River Estuary.
- Results are relevant to US-Mexico border communities and other polluted estuaries.
