# Supplementary material for: Evaluation of real-time fluorescence sensors and benchtop fluorescence for tracking and predicting sewage contamination in the Tijuana River Estuary at the US-Mexico border
Source: Sci Total Environ. Author manuscript; Available in PMC 2025 Nov 10. (PMC11629095; doi:10.1016/j.scitotenv.2024.175137)
Supplement: Supplement1 [file NIHMS2037805-supplement-Supplement1.pdf]

Supplemental Information

Evaluation of real-time fluorescence sensors and benchtop fluorescence for tracking and predicting sewage contamination in the Tijuana River Estuary at the US-Mexico border

Natalie Mladenov[1]*, Trent Biggs[2], Keyshawn Ford[1], Stephany Garcia[2], Yongping Yuan[3], Alexandra Grant[1], Elise Piazza[2], Elisa Rivera[1], Federick Pinongcos[1♦], Scott P. Keely[4], Callie Summerlin[2], Douglas Liden[5]

[1] *Department of Civil, Construction, and Environmental Engineering, San Diego State University, 5500 Campanile Drive, San Diego, CA 92182, United States of America*
[2] *Department of Geography, San Diego State University, 5500 Campanile Drive, San Diego, CA 92182, United States of America*
[3] *US Environmental Protection Agency, Office of Research and Development, 109 T. W. Alexander Dr., Research Triangle Park, NC 27711*
[4] *US Environmental Protection Agency, Office of Research and Development, 26 W. Martin Luther King Dr., Cincinnati, OH 45268*
[5] *United States Environmental Protection Agency-Region 9 Water Division, San Diego, CA*

[♦]Current affiliation: Trussell Technologies, San Diego, CA 92121

Supplemental Tables: 2

Supplemental Figures: 8

Supplemental Tables

Table S1. Untreated wastewater used in wastewater addition experiments: sampling dates and characteristics*.

Date collected	Specific conductivity (mS/cm)	pH	DOC (mg/L)	TDN (mg/L)	COD (mg/L)	Obtained from	PB CILA status**
Wastewater							
21 Jan 2022	NM	NM	27.6	70.0	247	SEWRF	N/A
1 Sep 2022	2.95	7.57	NP	NP	NP	IWTP	Operating
27 Oct 2022	9.69	7.49	159	44.0	186	IWTP	Pump down
17 Nov 2022	2.47	7.32	74.6	32.1	487	IWTP	Pump down
26 Oct 2023	2.43	7.44	43.8	51.9	641	IWTP	Pump down

* NM = not measured; NP = not preserved and therefore could not be measured; SEWRP = San Elijo Water Reclamation Facility; IWTP = International Water Treatment Plant; N/A = not applicable.

** Under "pump down" conditions, the international boundary pump station (PB CILA) was inoperational, and IWTP received only water from Tijuana's sanitary sewer. When PB CILA was operating, the IWTP treated water from both the Tijuana River and Tijuana's sanitary sewer. The SEWRF sample is untreated influent from a water reuse facility in San Diego County, USA; therefore pump status does not apply to this sample.

Table S2. Description of water quality analyses performed, equipment used, and preservation technique.

**Analysis**	**Instrument/ equipment used**	**Preservation and storage**	**Quality control measures**
pH and conductivity	Mettler Toledo SG23 pH/EC meter	analyzed on unfiltered samples on same day as sample retrieval	Measurements recorded in triplicate and standard deviations were within 10% of mean.
Total coliforms and *E. coli*	IDEXX Colilert-18 and Quantitrays	analyzed on unfiltered samples on same day as sample retrieval	DI field blank, filled in field and analyzed in the same way as collected samples. No field blank wells were yellow or fluorescent.
Enterococcus	IDEXX Enterolert and Quantitrays	analyzed on unfiltered samples on same day as sample retrieval	DI field blank, filled in field and analyzed in the same way as collected samples. No field blank wells were fluorescent.
Chemical oxygen demand (COD)	HACH kits and HACH DR3900 Spectrometer	addition of hydrochloric acid to pH 2 to unfiltered samples and stored refrigerated until analysis	Measurements recorded in triplicate and standard deviations were within 10% of mean. 15% of samples were run in duplicate and standard deviations were required to be within 10% of the mean.
Dissolved organic carbon (DOC) and total dissolved nitrogen (TDN)	Shimadzu TOC-L Total Organic Carbon and Total Nitrogen Analyzer	samples filtered through combusted glass fiber filter (0.7 µm nominal pore size); addition of hydrochloric acid to pH 2 and stored refrigerated until analysis	Each measurement is an average of 3 readings with coefficient of variability of < 20%. Duplicates were run for 15% of samples, and standard deviations were required to be within 10% of the mean.
3D excitation-emission matrix (EEM) fluorescence	Horiba Aqualog Spectro-fluorometer	samples filtered through combusted glass fiber filter (0.7 µm nominal pore size); analyzed on same day as sample retrieval.	Cuvette checked for contamination prior to spectral acquisition. Each EEM spectrum checked manually.

Supplemental Figures

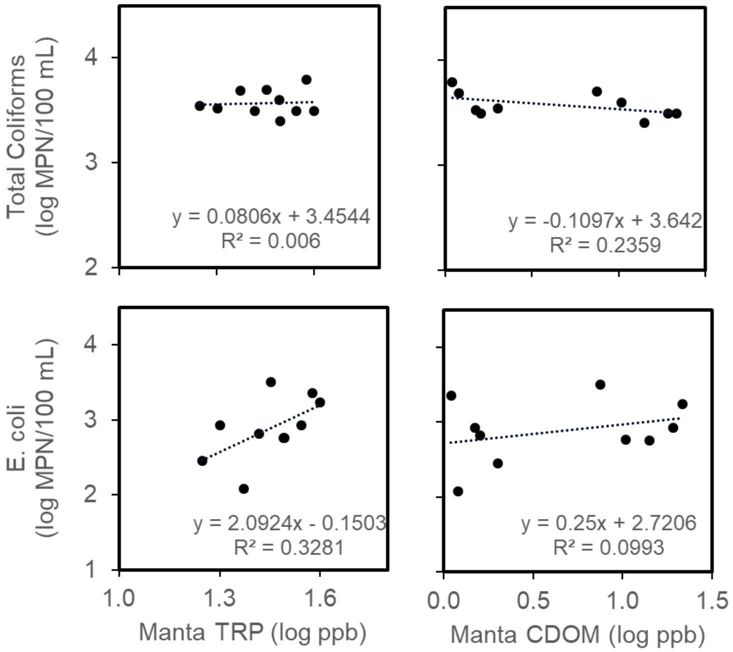

Figure S1. Correlations between fluorescence intensities of the in-situ (Manta) fluorometer and fecal indicator bacteria for samples collected during dry weather without cross-border flow on 24-25 May 2021. Benchtop fluorescence and enterococcus concentrations were not measured on this date.

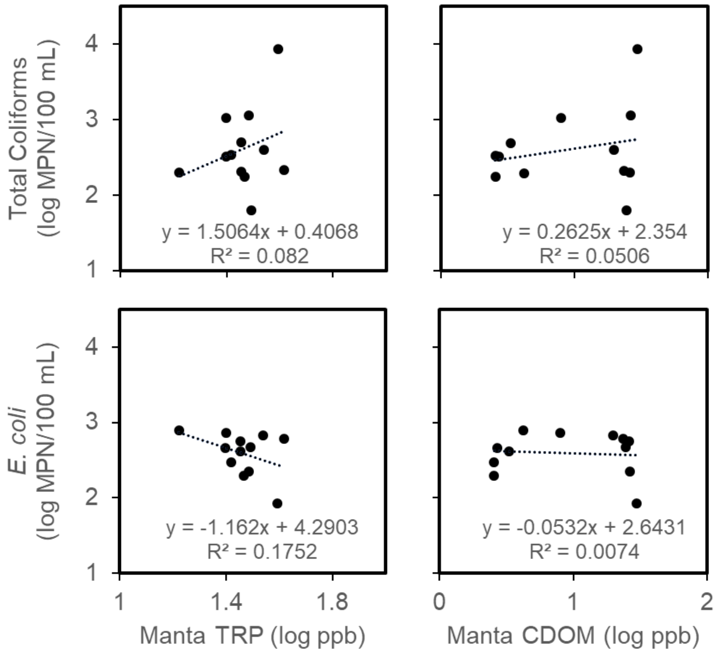

Figure S2. Correlations between fluorescence intensities of the in-situ (Manta) fluorometer and fecal indicator bacteria for samples collected during dry weather without cross-border flow on 6-7 June 2021. Benchtop fluorescence and enterococcus concentrations were not measured on this date.

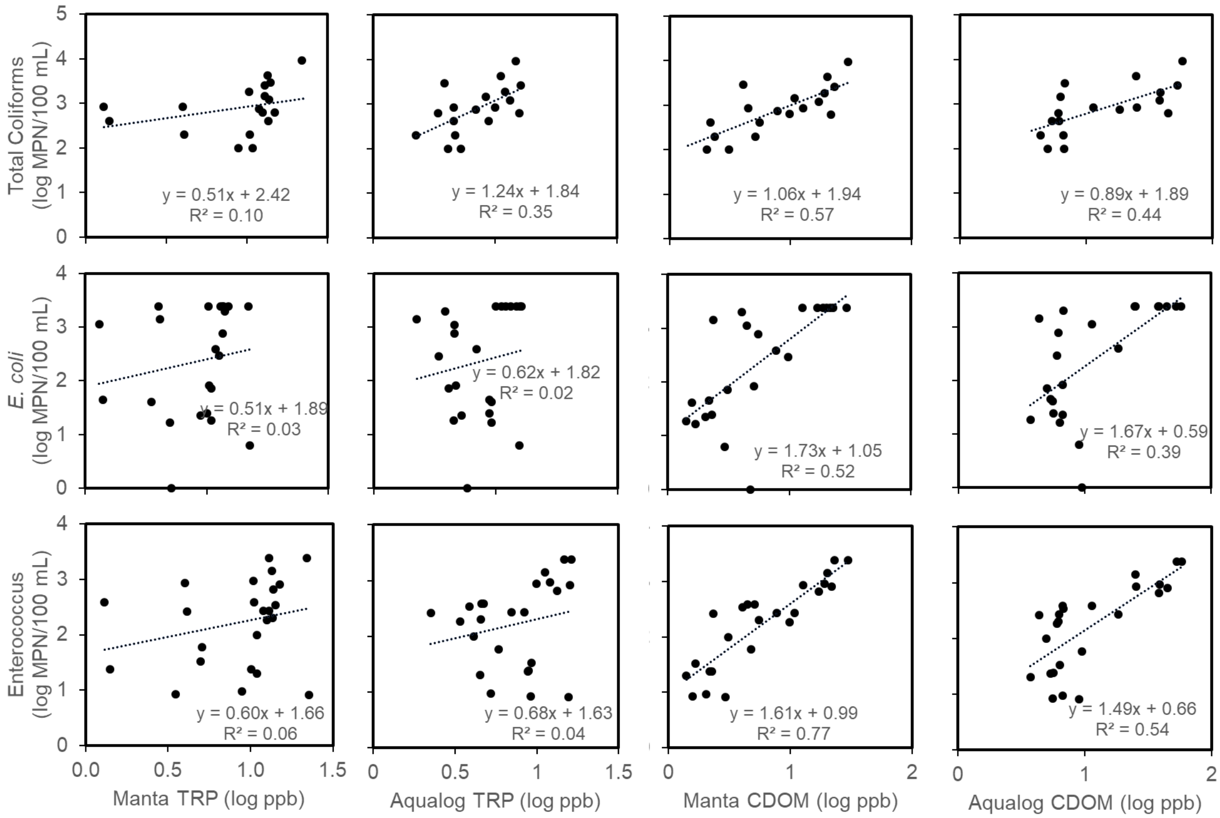

Figure S3. Correlations between fluorescence intensities of the in-situ (Manta) and benchtop (Aqualog) fluorometers and fecal indicator bacteria for samples collected during dry weather without cross-border flow in 11-12 September 2021.

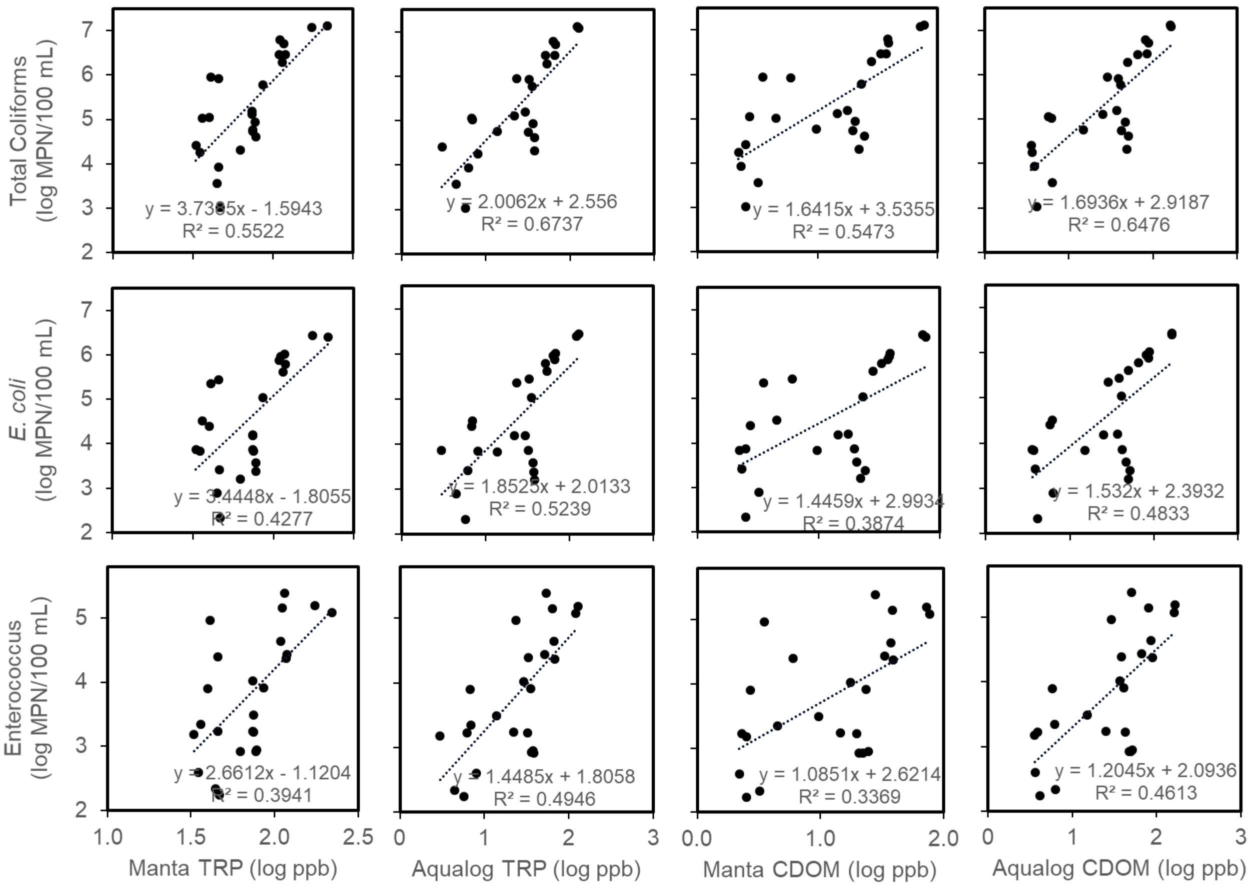

Figure S4. Correlations between fluorescence intensities of the in-situ (Manta) and benchtop (Aqualog) fluorometers and fecal indicator bacteria for samples collected during dry weather with cross-border flow in 3-4 March 2022. All bivariate correlations were significant ($p < 0.05$).

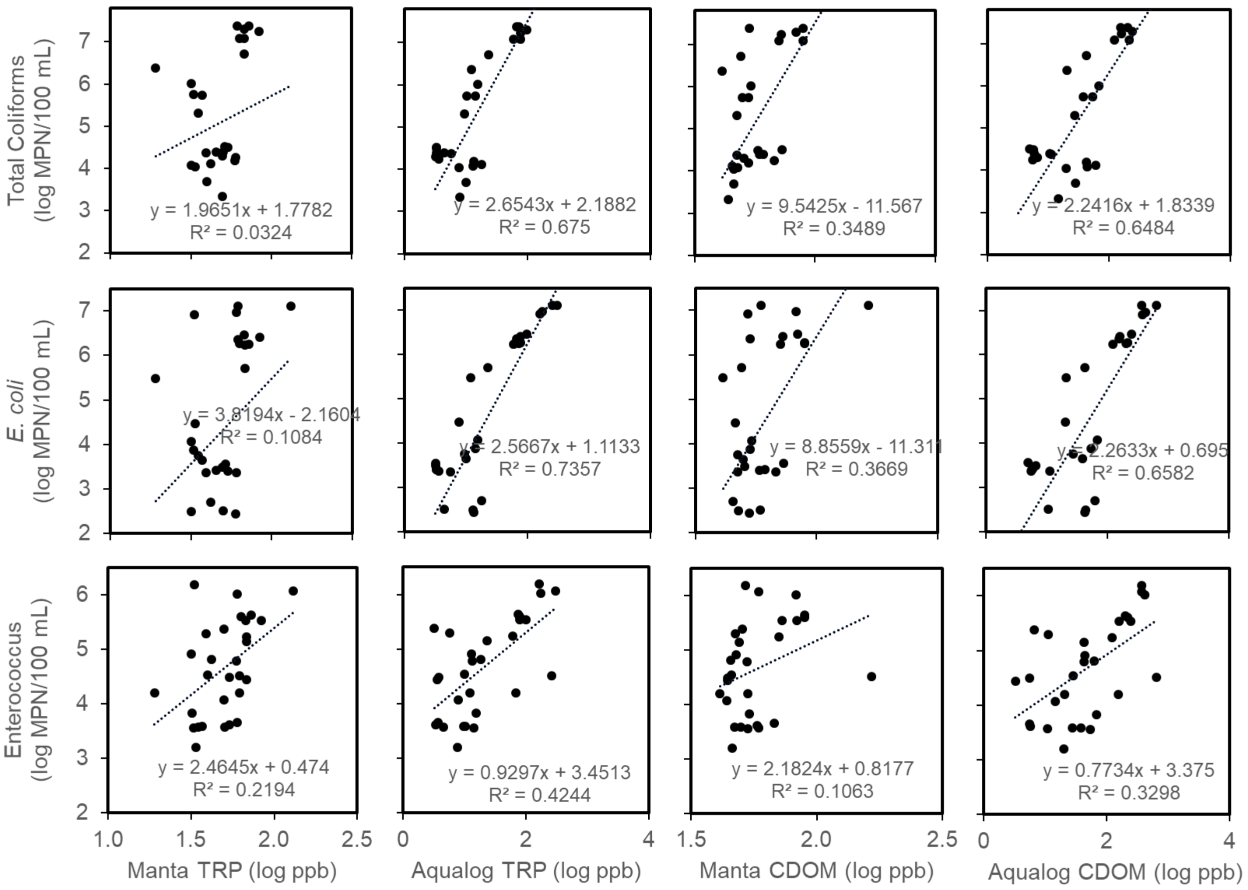

Figure S5. Correlations between fluorescence intensities of the in-situ (Manta) and benchtop (Aqualog) fluorometers and fecal indicator bacteria for samples collected during storm with cross-border flow in 9-10 September 2022. All bivariate correlations were significant ($p < 0.05$).

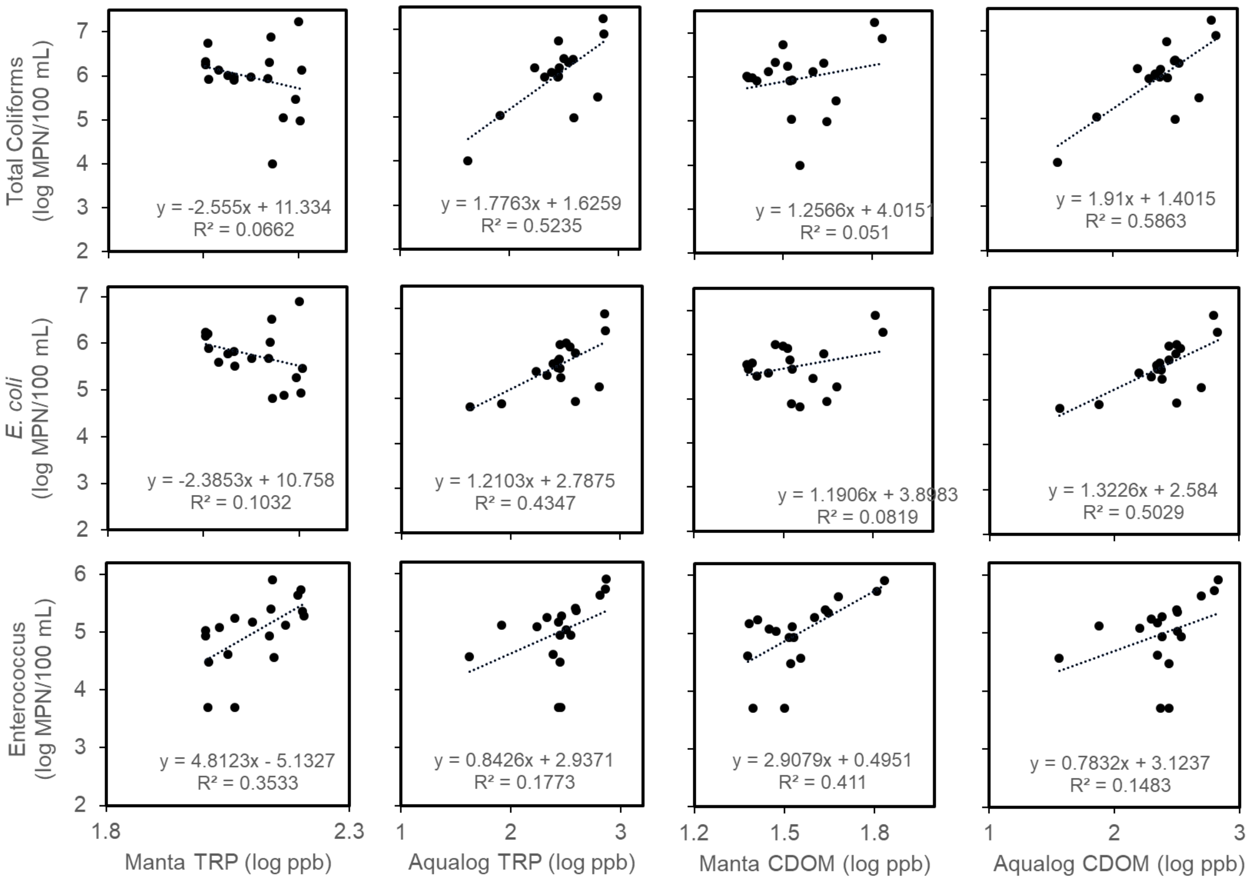

Figure S6. Correlations between fluorescence intensities of the in-situ (Manta) and benchtop (Aqualog) fluorometers and fecal indicator bacteria for samples collected during dry weather with cross-border flow in 7-8 September 2023.

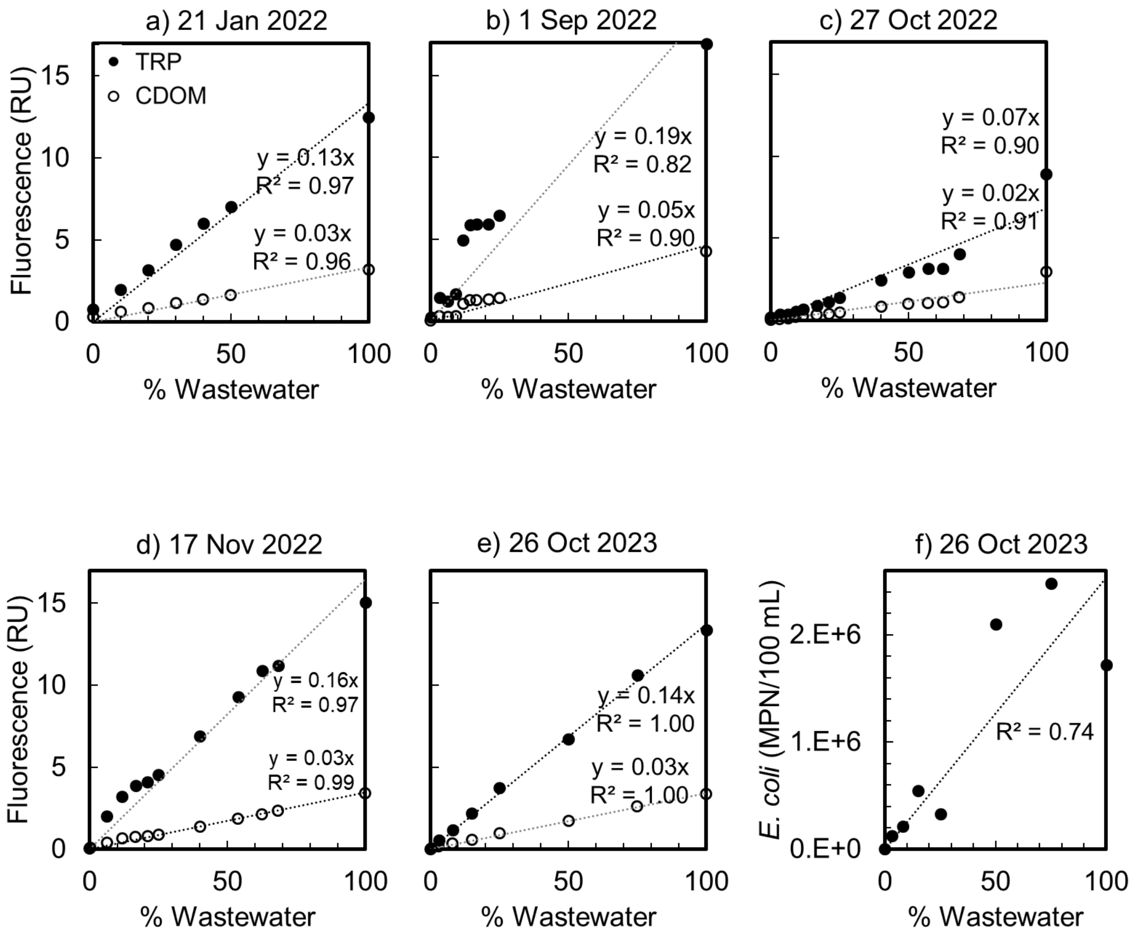

Figure S7. Scatterplots of % untreated wastewater added to seawater and benchtop fluorescence intensities of tryptophan-like (TRP) and humic (CDOM) peaks on five different dates (a through e). Panel (f) shows the relationship between *E. coli* and % wastewater for the 26 October 2023 wastewater addition experiment. Relationships are significant ($p < 0.01$) for all five experiments.

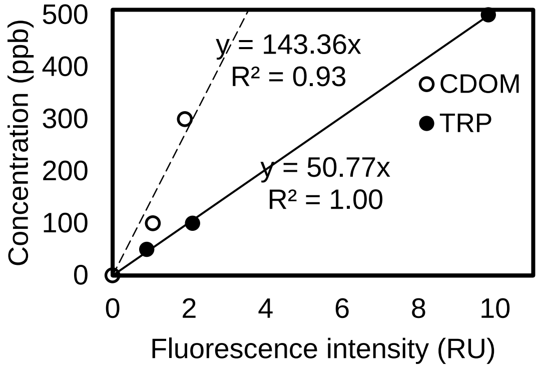

Figure S8. Calibration curve to relate Raman units (RU) to ppb tryptophan and ppb CDOM from calibrations with tryptophan and quinine sulfate standards, respectively, measured on the benchtop fluorometer on 26 October 2023.
